# Greater hip internal rotation range of motion is associated with increased dynamic knee valgus during jump landing, both before and after fatigue

**DOI:** 10.1002/ksa.12447

**Published:** 2024-08-27

**Authors:** Sandro Hodel, Florian B. Imhoff, Gerda Strutzenberger, Daniel Fitze, Simone Obrist, Lazaros Vlachopoulos, Johannes Scherr, Sandro F. Fucentese, Stefan Fröhlich, Jörg Spörri

**Affiliations:** ^1^ Department of Orthopaedics, Knee Surgery, Balgrist University Hospital University of Zurich Zurich Switzerland; ^2^ Department of Orthopaedics, Sports Medical Research Group, Balgrist University Hospital University of Zurich Zurich Switzerland; ^3^ Department of Orthopaedics University Centre for Prevention and Sports Medicine, Balgrist University Hospital, University of Zurich Zurich Switzerland

**Keywords:** anterior cruciate ligament (ACL), injury, prevention, sport

## Abstract

**Purpose:**

The aim of this study was to analyse sex‐specific differences contributing to dynamic valgus in competitive soccer players before and after a standardised fatiguing protocol.

**Methods:**

Thirty‐nine healthy female and male competitive soccer players (19 females and 20 males) were recruited for the purpose of this study. Bilateral medial knee displacement (MKD) was assessed during drop jump landings using a three‐dimensional motion capture system before and after a standardised fatiguing protocol. In addition, all soccer players underwent clinical examinations, including rotational hip range of motion (ROM), isokinetic strength testing and magnetic resonance imaging (MRI) of the hip and knee. Sex‐specific and fatigue‐dependent differences were reported, and the influence of demographic, clinical and radiographic factors on MKD was analysed via multiple linear regression models.

**Results:**

Compared with male soccer players, female soccer players demonstrated a tendency towards increased MKD during drop jump landings before (*p* = 0.09) and after the fatiguing protocol (*p* = 0.04). Sex‐specific differences included increased hip internal rotation (IR) ROM, decreased hip external rotation (ER) strength and increased femoral torsion in females (all *p* < 0.002). According to the multiple linear regression models (stepwise method), increased hip IR ROM (90° of flexion) and the non‐dominant leg remained the sole independent predictors of increased MKD during drop jump landings before (*p* < 0.01 and *p* = 0.02, respectively) and after fatigue (*p* < 0.01 and *p* < 0.01, respectively). An increase in hip IR ROM in females was linearly related to MKD after fatigue (*R* ^2^ = 0.25; *p* < 0.01).

**Conclusion:**

Female soccer players exhibited increased dynamic valgus before and after fatigue, which is likely attributed to joint mobility, as well as muscular and anatomical differences, such as increased hip IR ROM, reduced hip ER strength and increased femoral torsion. In particular, females with increased hip IR ROM were more susceptible to effects of fatigue on MKD, which may increase their risk for anterior cruciate ligament injury.

**Level of Evidence:**

Level III.

**Abbreviations**
3D: three‐dimensional
AASA: anterior acetabular sector angle
ACL: anterior cruciate ligament
DJ: drop jumps
ER: external rotation
IR: internal rotation
LCEA: lateral centre edge angle
LSI: limb symmetry index
MKD: medial knee displacement
MRI: magnetic resonance imaging
NLA/NLB: National League A/B
ROM: range of motion
SFL: Swiss Football League
VIF: variance inflation factor

## INTRODUCTION

Non‐contact anterior cruciate ligament (ACL) injuries are associated with biomechanically unfavourable movements of the knee in which the ACL is maximally tensioned, such as dynamic knee valgus and tibial internal or external rotation (ER) [20]. Especially, competitive soccer players are at risk for ACL injuries [6], and risk factors for non‐contact ACL injuries can be classified as environmental, hormonal, morphological or neuromuscular [8, 26]. In particular, the contributions of the two latter classes of factors to dynamic knee valgus are largely unexplored.

In general, female athletes are at increased risk of sustaining ACL injuries when adjusting for sports participation, activity level and exposure [25, 26]. Sex‐specific anatomical variations might play a role in determining the risk of ACL injury due to unfavourable lower extremity kinematics, such as extensive dynamic knee valgus. For example, higher grades of femoral torsion lead to an increase in the internal–rotation–adduction moment of the knee [9], resulting in an increase in the dynamic q‐angle and dynamic knee valgus [13]. Similarly, decreasing acetabular coverage may increase passive hip internal rotation (IR) and promote dynamic knee valgus patterns. In this context, medial knee displacement (MKD) has previously been used as a reliable proxy measure for dynamic knee valgus in cohorts of alpine skiers [11, 12], soccer players [27] and handball players [23]. As such, the MKD describes the medial directed movement of the knee joint centre, projected onto the anatomical frontal plane [12] and is a surrogate for the multiplanar dynamic valgus movement resulting from internal hip rotation and knee abduction that occur during drop jump (DJ) landings. In addition, fatigue may influence MKD and thus increase the risk of ACL injury [13].

Accordingly, sex‐specific and fatigue‐dependent factors could potentially amplify movement alterations and proprioceptive acuity, and female soccer players may be at increased risk of ACL injury due to their distinct dynamic alignment, which is affected by particular bony anatomy, morphological hip and thigh muscle properties, hip muscle strength, core stability, hip and thigh muscle activation patterns and individual levels of fatigue.

The aim of the present study was to identify sex‐specific differences contributing to MKD motion in female and male competitive soccer players before and after a standardised fatiguing protocol. It was hypothesised that valgus loading during DJs is greater in female soccer players than in males due to anatomical variances in hip joint anatomy and decreased muscle strength at the lower extremities. Second, it was hypothesised that this effect would be more pronounced after a fatiguing protocol.

## MATERIALS AND METHODS

### Participants and study design

Thirty‐nine healthy competitive soccer players (19 females and 20 males) were included in this experimental, cross‐sectional study. The inclusion criteria were active participation in the Swiss National League A and B (NLA/NLB) (females) or in the Swiss Football League (SFL) (males) corresponding to a Tegner score ≥9 [29]. The exclusion criteria were previous hip, knee or ankle surgeries; prior ACL ruptures or chronic pain at the hip, knee or ankle. The demographic characteristics of the participants are presented in Table 1. All participants underwent various evaluations, including clinical assessments, hip and thigh muscle strength testing, radiographic assessment of hip and knee anatomy and three‐dimensional (3D) motion capture while performing DJs before and after the implementation of a standardised fatiguing protocol.

**Table 1:** Demographic characteristics of the soccer players.

Charactertistics	Females (*n* = 19, 48.7%)	Males (*n* = 20, 51.3%)	Total (*n* = 39, 100%)
Age (years)	21.7 ± 3.3	22.0 ± 2.7	21.9 ± 3.1
Height (cm)	166.3 ± 6.6	178.2 ± 6.3	172.4 ± 8.8
Weight (kg)	62.9 ± 8.6	73.9 ± 7.9	68.5 ± 10.0

### Clinical assessments and ROM

Pre‐existing pathologies and the Tegner score were identified via clinical examination by one experienced sports medicine physician. The hip range of motion (ROM), including IR and ER, was assessed while the subjects were in the supine position at 90° of hip flexion using a goniometer. Bilateral analysis was performed for all soccer players, resulting in 78 legs for analysis.

### Hip muscle strength

An isokinetic dynamometer (Con‐Trex MJ; CMV AG) was used to assess muscle strength. After a warm‐up and practice session, concentric and eccentric hip rotational forces were measured with an isokinetic dynamometer on both sides according to the manufacturer's guidelines. All participants were placed in a supine position with the hip and knee in a neutral position. When investigating the potential associations between MKD during DJ landing and external hip rotator strength, it is crucial to test it in similar positions; this means mimicking the relatively neutral hip and knee positions that are quality criteria for vertical DJs. In addition, when assessing peak torques in an eccentric contraction mode, non‐neutral hip and knee positions may expose the knee to adverse loadings; accordingly, we decided explicitly for a supine position. The axis of the dynamometer was aligned with the axis of the femur. Concentric and eccentric angular velocities at 60°/s were used. The peak torque (Nm) at each velocity was evaluated for IR and ER.

### Radiographic assessment of hip and knee anatomy

All participants underwent a standardised magnetic resonance imaging (MRI) protocol of the hip and knee using 1.5 mm thick slices with coronal, sagittal and axial reconstructions (T1‐ and T2‐weighted images). We sought to investigate the anatomical factors that may influence hip ROM and MKD motion, including acetabular coverage and femoral torsion. Therefore, the following radiographic parameters were assessed via MRI: femoral torsion [28], lateral centre edge angle (LCEA) [14], anterior acetabular sector angle (AASA) [7] and alpha angle [14]. The measurements were performed by a blinded orthopaedic surgeon and a musculoskeletal radiologist.

### 3D motion capture while performing DJs before and after the implementation of a standardised fatiguing protocol

All participants underwent MKD testing during DJs before and after a standardised fatiguing protocol. For this purpose, participants performed DJs from a box with a height of 32.6 cm. All participants were instructed to jump as high as possible with as short ground contact as possible after the drop. The 3D motion patterns were assessed using an optoelectronic 3D motion capture system with 14 infrared cameras (Vero v2.2; Vicon, Oxford Metrics, 200 Hz) and 38 reflective markers and four marker clusters according to the Cleveland Clinical Markerset. Kinetic data of the ground contact for the right and left limbs were measured by the use of two 3D force plates (40 × 60 cm; Kistler 9260AA6, Kistler Holding AG, 2000 Hz) embedded in the ground. All measurements and post‐processing steps were conducted as described in detail in Strutzenberger et al. [27]. The primary kinematic outcome of MKD [1] before and after fatiguing was determined in accordance with the definitions of Ellenberger et al. [12]. First, the contact phase was determined as the time period, in which the raw signal of the vertical force exceeded 25 N. Second, a reference plane connecting the hip, knee joint centre and ankle joint centre was defined as one frame (0.005 s) before force plate contact and remained fixed in the hip joint centre. The MKD was then calculated as the medial distance (in mm) between the knee joint centre and the reference plane throughout the contact phase. Subsequently, the maximum MKD during the contact phase was detected and used for further analysis. A high MKD represents high out‐of‐plane movement and serves as a proxy for the combination of increased dynamic knee valgus and internal hip rotation. The point determination accuracy using Vicon systems has been demonstrated to be less than 1 mm for dynamic measurements at speeds >3 m/s [22]. In a previous study using the same methodology, the test‐retest reliability of the 3D motion capture‐based determination of MKD during DJ landings was reported to be *good* (ICC (3,1) = 0.83) [12].

All the subjects underwent a standardised fatiguing protocol. The fatiguing protocol was adapted from Fidai et al. [13] to include both muscular and cardiovascular fatigue and was already described in detail by Strutzenberger et al. [27]. In brief, the protocol consisted of multiple repetitions of a task cycle including 60 s jumping jacks, 30 s squats with a 180° jump turn, 60 s spiderman planks, 30 s alternating lunge jumps, 60 s alternating side lunges and 30 s sumo squats with leg curls.

After each cycle, participants were asked to rate their subjectively perceived level of fatigue based on a visual analogue scale (VAS) (0: not fatigued at all, 10: extremely fatigued). The fatiguing protocol was continued until, after a minimum of four cycles, the VAS score was (i) eight twice in a row or (ii) one time ten. Lactate levels were measured based on blood samples (Lactate Scout 4; EKF Diagnostics, SensLab GmbH) to quantify the effect of the fatiguing protocol. On average, blood lactate levels increased from 0.7 ± 0.3 to 9.4 ± 2.6 mmol/L from pre‐workout to direct post‐workout (*p* < 0.001).

### Sample size calculation

Assuming that a meaningful difference in the primary outcome variable (MKD) is based on an effect size of Cohen *d* = 0.864, as found in Ellenberger et al. [12] for sex differences in elite athletes during single‐leg squats and based on *α* = 0.05 and 1 − *β* = 0.80, an a priori power analysis yielded a required sample size of approximately *n* = 46.

### Ethical statement

The underlying study protocol was approved by an institutional review board and the local ethics committee (KEK‐ZH‐NR: 2020–02583). Before participation, all the subjects agreed and provided written informed consent. All procedures were in accordance with the Declaration of Helsinki and national laws.

### Statistical analysis

Normality was tested using the Shapiro–Wilk test. All the data are presented as the means and standard deviations (SDs) or as counts and percentages. The increase in lactate was tested for significance using a paired Wilcoxon test. Sex differences were analysed using non‐paired sample *t* tests or Mann‒Whitney *U* tests, as appropriate, or Chi‐Square tests for categorical variables. A multiple linear regression model (stepwise method) was used to assess the influence of the following predictors of MKD at rest and at fatigue: demographic factors (sex, height, weight, leg dominance and hip ROM), radiographic factors (femoral torsion, LCEA, AASA and alpha angle) and strength (concentric and eccentric hip ER peak force). Multicollinearity was assessed using the variance inflation factor (VIF), which was deemed acceptable for all variables (VIF < 5). Regression coefficients are reported as *β* (95% confidence interval [CI]), and model fit is reported as *R* ^2^. All the statistical analyses were performed using SPSS statistical software (v.20). The significance was set at *p* < 0.05.

## RESULTS

**Figure 1:**
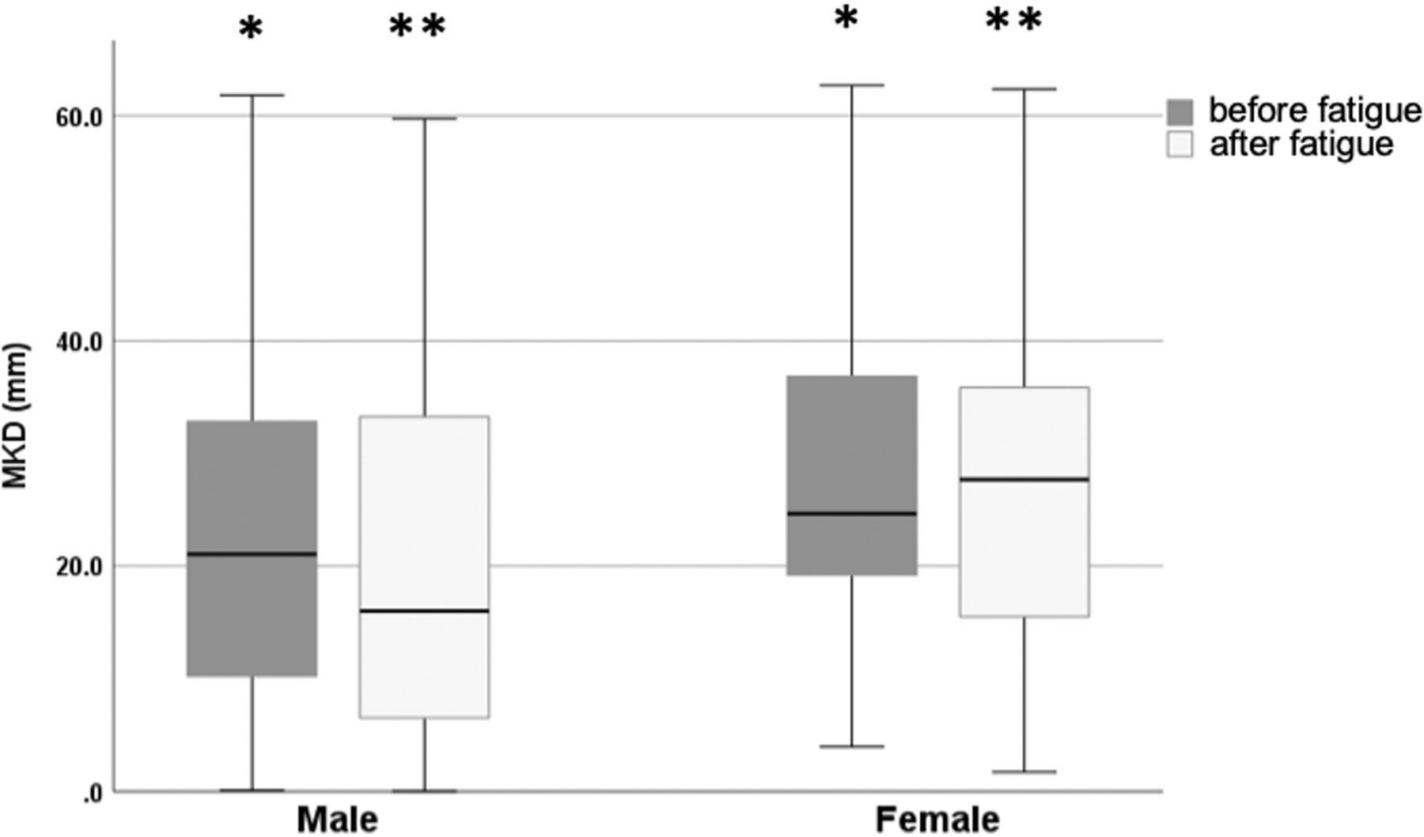
Medial knee displacement (MKD) before and after the fatiguing protocol according to sex: Female soccer players demonstrated a tendency for an increased MKD during a drop jump before (*p* = 0.09)* and after the fatiguing protocol (*p* = 0.04)**. Boxplots depict medians (lines), interquartile ranges (boxes) and maximum values (whiskers).

Compared to male soccer players, female soccer players demonstrated increased MKD during DJs, with a non‐significant tendency before fatigue (*p* = 0.09) and a significant difference after the fatiguing protocol (*p* = 0.04) (Figure 1 and Table 2).

**Table 2:** Medial knee displacement (MKD) during drop jumps before and after the fatiguing protocol according to sex.

Characteristics	Female	Male	*p*‐Value
	*n* = 19	*n* = 20	
MKD (mm)			
Rest	30 ± 18	23 ± 16	0.09
Fatigue	28 ± 15	21 ± 18	**0.04**
Difference	−2 ± 13	−2 ± 8	0.83
Maximum knee flexion (°)			
Rest	66 ± 8	66 ± 10	0.85
Fatigue	64 ± 7	64 ± 10	0.70
Difference	−2 ± 7	−3 ± 4	0.15
Increase in MKD after fatiguing *n* (%)	15 (39.5)	15 (37.5)	0.52

*Note*: Significant *p* values are marked in bold.

Sex‐specific differences included increased hip IR ROM, decreased hip ER strength and increased femoral torsion in females (all *p* < 0.01) (Table 3).

**Table 3:** Sex‐specific differences in bony morphology and hip isokinetic strength.

Characteristics	Female	Male	*p*‐Value
	*N* = 19	*N* = 20	
Height (cm)	166.3 ± 6.7	178.2 ± 6.5	**<0.01**
Weight (kg)	62.9 ± 8.9	74.1 ± 7.9	<**0.01**
Leg dominance			
Right	18 (94.7)	19 (95)	0.49
Left	1 (5.3)	1 (5)	
Hip ROM (°)			
Supine (90° flexion)			
IR	35.9 ± 7.1	26.9 ± 7.8	<**0.01**
ER	42.0 ± 7.6	39.1 ± 7.2	0.09
Radiographic parameters (°)			
Acetabular			
AASA (°)	61.4 ± 8.1	59.8 ± 5.1	0.16
LCEA (°)	28.5 ± 6.2	26.4 ± 5.6	0.06
Femoral			
Femoral torsion (°)	24.9 ± 6.2	19.2 ± 8.5	**<0.01**
Alpha angle (°)	48.6 ± 7.8	60.5 ± 9.6	<**0.01**
Maximum hip external rotation peak force (Nm) (60 °/s)			
Concentric	26.6 ± 5.7	37.2 ± 11.7	<**0.01**
Eccentric	33.2 ± 6.9	45.1 ± 10.2	<**0.01**

*Note*: Significant *p* values are marked in bold.

Abbreviations: AASA, anterior acetabular sector angle; ER, external rotation; IR, internal rotation; LCEA, lateral centre edge angle; ROM, range of motion.

According to the multiple linear regression models (stepwise method), increased hip IR ROM and leg dominance remained the sole significant predictors of MKD before fatigue (*β* = 0.7; 95% CI: 0.2–1.0; *p* < 0.01; and *β* = 8.3; 95% CI: 2.7–16.5; *p* = 0.02, respectively); model fit: *R* ^2^ = 0.18. These predictors remained significant after the fatiguing protocol (*β* = 0.6; 95% CI: 0.3–1.1; *p* < 0.01; and *β* = 9.6; 95% CI: 1.2–15.4; *p* < 0.01; model fit: *R* ^2^ = 0.19).

**Figure 2:**
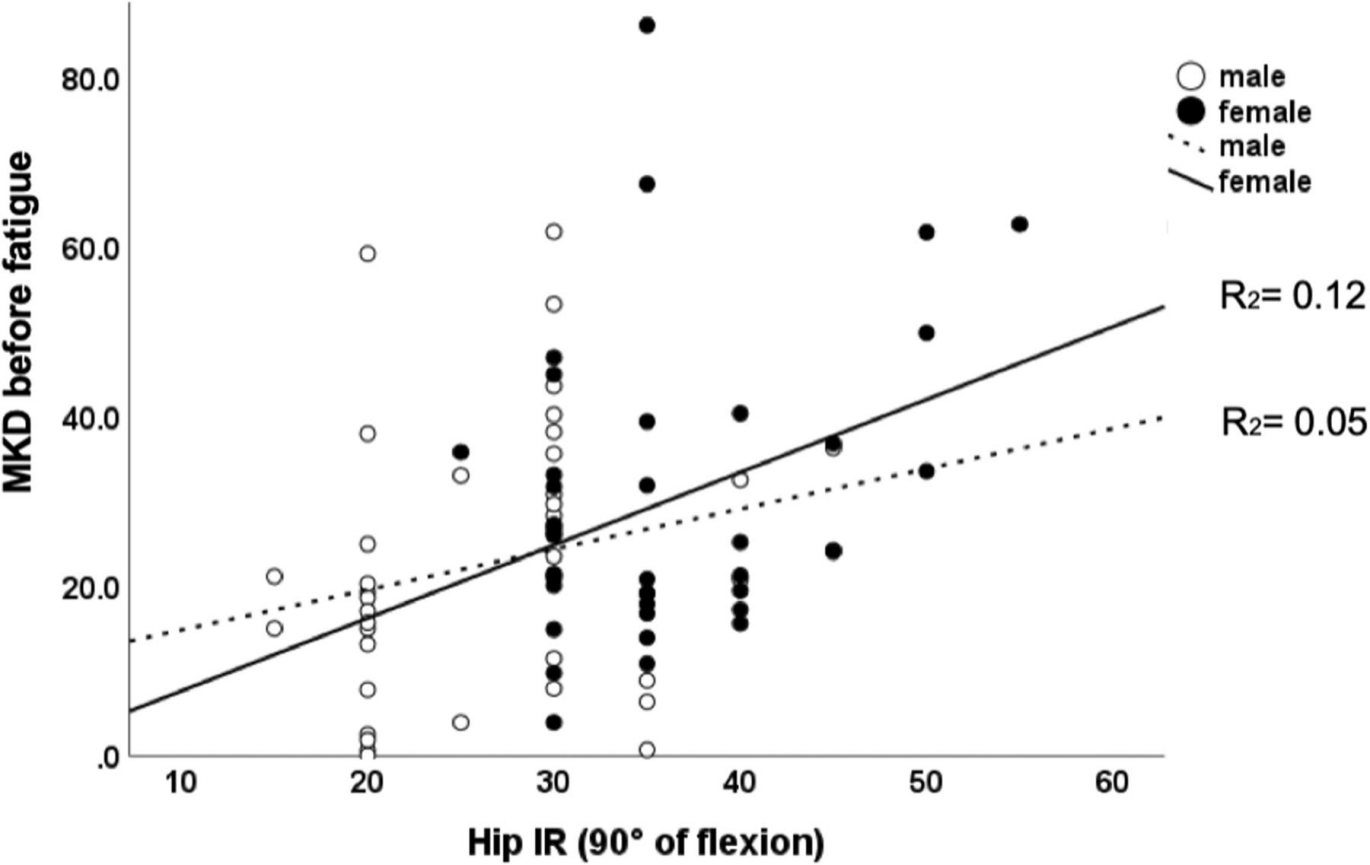
Scatterplot depicting the linear relationship between hip internal rotation (IR) range of motion (ROM) and medial knee displacement (MKD) in female (*R* ^2^ = 0.12; *p* = 0.03) and male (*R* ^2^ = 0.05; *p* = 0.15) soccer players at rest.

**Figure 3:**
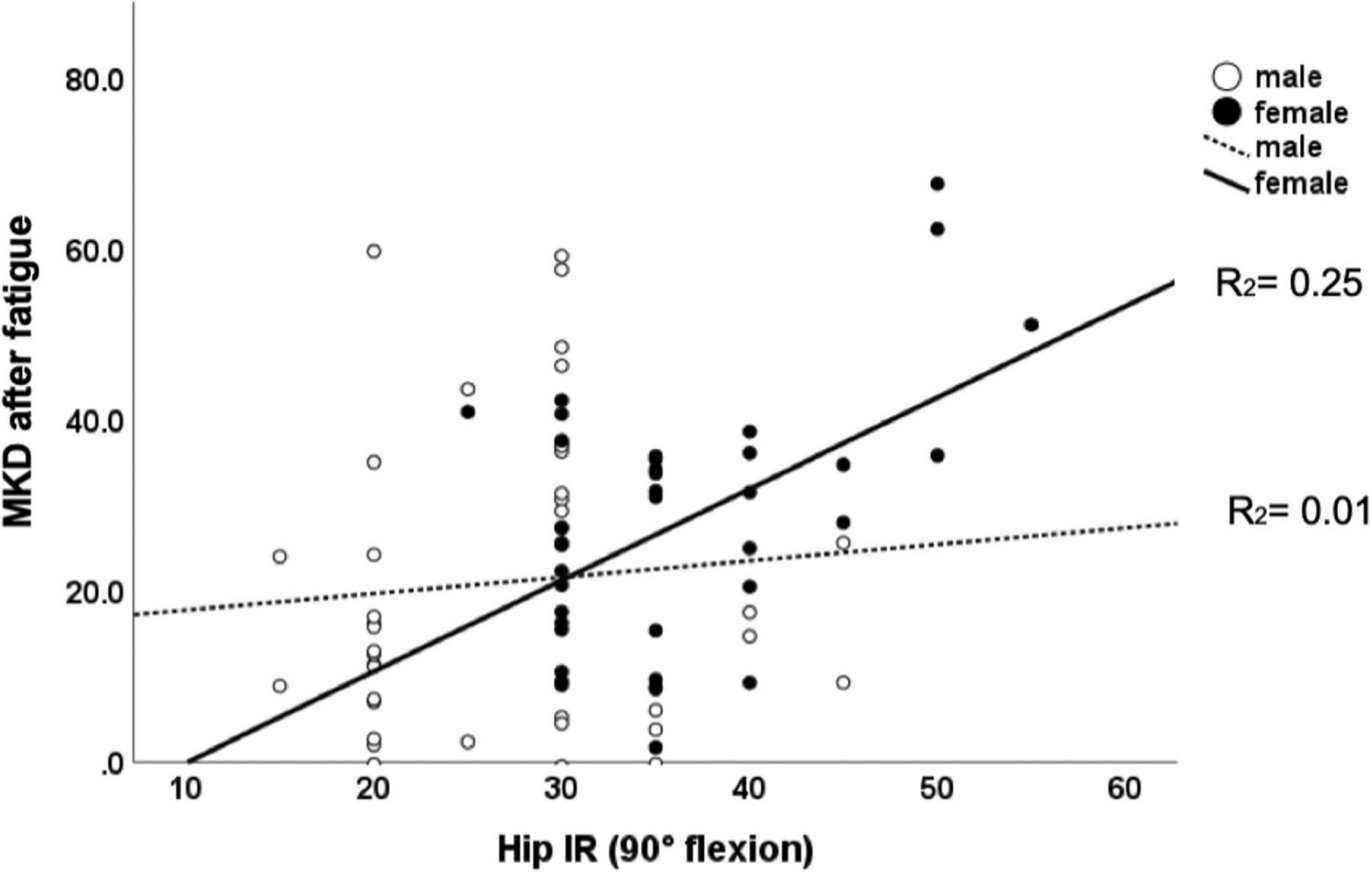
Scatterplot depicting the linear relationship between hip internal rotation (IR) range of motion (ROM) and medial knee displacement (MKD) in female (*R* ^2^ = 0.25; *p* < 0.01) and male (*R* ^2^ = 0.01; *p* = 0.60) soccer players after the fatiguing protocol.

The linear effect of hip IR ROM at 90° of flexion on MKD could only be demonstrated in female soccer players but not in male soccer players, and this effect was more pronounced after fatiguing (Figures 2 and 3).

The non‐dominant leg demonstrated an increased MKD (30 ± 18 mm) compared to that of the dominant leg before (23 ± 16 mm; *p* = 0.045) and after the fatiguing protocol (29 ± 18 mm; vs. 20 ± 15 mm; *p* = 0.045). Hip ER strength did not differ between the dominant and non‐dominant legs during DJs (n.s.).

## DISCUSSION

The main findings of this study are that compared with their male counterparts, female soccer players demonstrated increased MKD both at rest and following a standardised fatiguing protocol, whereas overall fatiguing did not increase MKD regardless of sex. Sex‐specific differences were identified, including increased hip IR ROM, decreased hip ER strength and increased femoral torsion in females.

While several studies have revealed that females are at increased risk for noncontact ACL injury due to hormonal, neuromuscular and anatomical factors [25, 26], the roles of hip anatomy, strength and fatiguing have been investigated only scarcely [13]. In line with previous results [24], our cohort revealed increased dynamic valgus in female soccer players. Anatomical sex‐specific differences were identified and included increased hip IR ROM and decreased hip ER strength. Increased hip IR ROM is mainly the combined result of anatomical differences and laxity. Increased femoral torsion and a decreased alpha angle allow for greater impingement‐free hip IR. An analysis of sex‐specific anatomical hip morphology and its influence on MKD has not yet been performed. In addition to an increased passive ROM, greater femoral torsion potentially leads to an unfavourable course of action for abductors and hip external rotators and a smaller lever arm [15, 17]. Overall, increased passive IR may put them at risk for increasing dynamic valgus loading patterns and maximising the load4 on the ACL, which is in accordance with previous results that linked femoral torsion to IR–adduction moments of the knee [4, 10, 18].

The susceptibility to the fatiguing protocol was independent of sex in this cohort, with approximately 40% of the soccer players demonstrating increased dynamic valgus after the fatiguing protocol. However, in female soccer players, a linear relationship between increasing hip IR and MKD, particularly after fatiguing, was demonstrated, in contrast to that in male soccer players. This finding questions a purely sex‐specific fatigue dependence but rather suggests confounding underlying contributing factors, such as increased IR ROM, decreased ER strength and increased femoral torsion. Moreover, this finding suggests that females with increased hip IR are at high risk for fatigue‐dependent noncontact ACL injury. Increased hip IR allows for increased femoral IR during landing in DJs and therefore favours a dynamic valgus landing pattern, subsequently increasing strain on the ACL and potentially leading to ACL rupture. However, the role of exercice‐induced fatigue after ACL reconstruction cannot be answered with this study but is likely to be affected in the same way or even accentuated as proprioceptive acuity decreases as demonstrated by Kim et al. [19].

Our data additionally showed that regardless of sex, there was an increase in MKD in the non‐dominant limb, suggesting an asymmetric landing strategy. While we discussed and investigated the sex‐specific anatomical variances leading to increased hip IR, leg dominance was found to be independent of sex. As no differences were detected between the dominant and non‐dominant legs, neuromuscular factors seem to be the reason for these findings, in accordance with Hanimann et al. [16]. It is possible that females are more strongly affected by these neuromuscular factors, as in female players, the non‐dominant limb is more prone to ACL injury [5], while male players tend to injure their ACL on the dominant limb [3]. Limb symmetry depends on leg dominance, as frequently assessed using the limb symmetry index (LSI) [30]. This could influence our findings; however, quadriceps and hamstring isokinetic strength was not assessed in our cohort.

The current study needs to be interpreted in light of its limitations. First, no reliability testing of dynamic valgus movement using the MKD was performed; however, the test‐retest reliability of this method has been shown to be good in a previous study [12]. Second, the fatiguing protocol most likely did not put every participant in a comparable fatigued state. However, to mitigate this potential bias, a standardised fatiguing exercise was applied, and increasing the blood lactate concentration significantly promoted adequate fatiguing. Third, MKD was assessed throughout the contact phase of landing and may not be exactly representative of the timing of injury, which has been reported to occur within 100 ms after initial foot contact [21] and also the clinical influence on functional performance tests or patient‐reported outcomes cannot be concluded from this study design [2]. Fourth, the number of participants in the current study was limited by the athletes' willingness to volunteer for this very time‐intensive study, which is why the current study may be slightly underpowered.

## CONCLUSION

Female soccer players exhibited increased dynamic valgus at rest and after fatiguing, which is likely attributed to joint mobility, as well as muscular and anatomical differences, such as increased hip IR ROM, reduced hip ER strength and increased femoral torsion. In particular, females with increased hip IR ROM were more susceptible to the effects of fatigue on MKD, which may increase their risk for ACL injury.

## AUTHOR CONTRIBUTIONS

Florian B. Imhoff, Stefan Fröhlich, Johannes Scherr, Sandro F. Fucentese and Jörg Spörri conceptualised and designed the study. Jörg Spörri recruited the participants and organised the data collection. Gerda Strutzenberger, Simone Obrist and Jörg Spörri collected the data. Sandro Hodel, Gerda Strutzenberger, Simone Obrist and Daniel Fitze processed the data and performed the statistical analysis. All authors substantially contributed to the interpretation of data. Sandro Hodel drafted the manuscript; all authors revised it critically, approved the final version of the manuscript and agreed to be accountable for all aspects of the work.

## CONFLICTS OF INTEREST STATEMENT

Sandro F. Fucentese is a consultant for Medacta SA (Switzerland), Zimmer Biomet (USA), Smith & Nephew (UK) and Karl Storz SE & Co. KG (Germany). Sandro F. Fucentese is a board member of the EKA‐ESSKA osteotomy expert group. The remaining authors declare no conflicts of interest.

## ETHICS STATEMENT

The underlying study protocol was approved by an institutional review board and the local ethics committee (KEK‐ZH‐NR: 2020–02583). All participants gave written informed consent to conduct and publish this study.

## Data Availability

No data repository online. Data are made available upon reasonable request.
